# The impact of primary school nutrition policy on the school food environment: a systematic review

**DOI:** 10.1093/heapro/daac084

**Published:** 2022-09-27

**Authors:** Lily Grigsby-Duffy, Ruby Brooks, Tara Boelsen-Robinson, Miranda R Blake, Kathryn Backholer, Claire Palermo, Anna Peeters

**Affiliations:** School of Health and Social Development, Deakin University, Geelong, Australia Global Obesity Centre (GLOBE), Institute for Health Transformation, Melbourne, VIC 3220, Australia; School of Health and Social Development, Deakin University, Geelong, Australia Global Obesity Centre (GLOBE), Institute for Health Transformation, Melbourne, VIC 3220, Australia; School of Health and Social Development, Deakin University, Geelong, Australia Global Obesity Centre (GLOBE), Institute for Health Transformation, Melbourne, VIC 3220, Australia; School of Health and Social Development, Deakin University, Geelong, Australia Global Obesity Centre (GLOBE), Institute for Health Transformation, Melbourne, VIC 3220, Australia; School of Public Health and Preventive Medicine, Monash University, Alfred Hospital, Commercial Road, Melbourne, VIC 3004, Australia; School of Health and Social Development, Deakin University, Geelong, Australia Global Obesity Centre (GLOBE), Institute for Health Transformation, Melbourne, VIC 3220, Australia; Department of Nutrition, Dietetics and Food, Monash University, Level 1, 264 Ferntree Gully Road, Notting Hill, VIC 3168, Australia; School of Health and Social Development, Deakin University, Geelong, Australia Global Obesity Centre (GLOBE), Institute for Health Transformation, Melbourne, VIC 3220, Australia; School of Public Health and Preventive Medicine, Monash University, Alfred Hospital, Commercial Road, Melbourne, VIC 3004, Australia

**Keywords:** nutrition policy, food environment, health equity, schools, children

## Abstract

School nutrition policies that aim to address unhealthy diets have been introduced in many countries. This systematic review aimed to synthesize the international literature to determine the impact (overall and by socioeconomic position [SEP]) of primary school nutrition policies on the availability of foods and beverages in schools. Seven databases were searched using keywords and medical subject headings related to nutrition policies and schools. Studies that reported on the impact of implemented school nutrition policies on food and beverage availability within primary schools were included. Eighteen studies (reported across 20 papers) were included. Fifteen of the included studies reported some positive impacts of policies, including increased availability of healthier foods and decreased availability of less healthy foods. Five studies focused specifically on schools in low-income communities and a further three specifically compared schools by SEP, with mixed findings. Two studies reported on factors influencing policy implementation, reporting a lack of financial resources as a barrier to schools offering a wider selection of healthy foods and additional school resources as increasing the likelihood of offering healthy foods. School nutrition policies appear to be effective at improving the healthiness of foods and beverages available at schools. Furthermore, the results suggest that well-implemented school nutrition policies that improve the healthiness of foods available are unlikely to exacerbate the socioeconomic gradient of poor nutrition. However, the number of studies that reported results by SEP limits drawing strong conclusions regarding equity impacts and we strongly recommend further studies analyze their findings according to SEP.

## INTRODUCTION

Globally, school-age children are under-consuming healthy foods such as fruits and vegetables and over-consuming unhealthy snacks (UNICEF, 2019). Over 18% of children (aged 5–19 years) were reported to be living with overweight or obese in 2016 (World Health Organization, 2020). Dietary habits in childhood predicts lifetime habits (UNICEF, 2019) making this an important time for establishing healthy habits. School food environments are promoted as a key setting for interventions to improve diet quality (World Health Organization, 1998, 2021). The school food environment refers to the availability, affordability, and promotion, of foods and beverages, served or sold inside and around the school premises, including, but not limited to, canteens, tuck shops and vending machines (Food and Agriculture Organization, 2019). This review focuses on one aspect of the school food environment: availability.

The World Health Organization has recommended the adoption of school nutrition policies that restrict the availability of less healthy foods and beverages (hereafter foods and beverages referred to as ‘food/s’) (World Health Organization, 2004, 2021). School nutrition policies can be voluntary or mandatory and vary in scope, from introducing nutrition education to the school curriculum to restricting unhealthy foods in vending machines. School nutrition policies tend to be government-directed while implementation responsibility usually sits internally within the school (World Cancer Research Fund International, 2022). School nutrition policies addressing the food environment have previously demonstrated increases in fruit and vegetable consumption and reductions in sugar-sweetened beverages, unhealthy snacks, fat, saturated fat and sodium intake (Micha *et al.*, 2018). Such policies have been adopted in many locations, at varying levels of governance, and with varying requirements for compliance (Storcksdieck Genannt Bonsmann, 2014; World Cancer Research Fund International, 2022). However, unless these policies are adopted and lead to changes in food availability, they will have limited ability to influence social norms around healthy eating and diet quality. Evaluating the impact policies have on the foods available within schools is important to better understand their implementation and feasibility as a strategy to improve children’s diet quality.

The impact of school nutrition policies on the school food environment has been partially explored in two systematic reviews. Both reviews found policies were generally associated with increased availability of healthier foods and/or decreased availability of less healthy foods (Jaime and Lock, 2009; Chriqui *et al.*, 2014). The first review (Jaime and Lock, 2009) only included studies in which policies had been adopted for the purposes of research trials; findings therefore may not reflect ‘real world’ policy implementation. Furthermore, the first review was published in 2009—many additional policy evaluations have since been published. The second review (Chriqui *et al.*, 2014), published in 2014, included only implemented policies but was limited to schools in the United States of America (USA) where the education system and school food services differ from that of other countries.

Neither of the previous reviews reported on the differential policy impact by school-level indicators of socioeconomic position (SEP). In high-income countries (including Australia, the United States of America and multiple countries across Europe), diet quality is generally lower, and the prevalence of diet-related diseases such as cardiovascular disease and cancer is higher, among those with lower SEP (Backholer *et al.*, 2016; Stringhini *et al.*, 2017; Chung *et al.*, 2018; Fismen *et al.*, 2021). Whilst in low- and middle-income countries, high SEP is associated with some healthier dietary patterns (e.g. higher consumption of fruits and vegetables) (Mayén et al., 2014), and a lower risk of many non-communicable diseases (e.g. cardiovascular diseases) (Sommer *et al.*, 2015), but also associated with unhealthy dietary patterns (e.g. higher intakes of calories, fat and processed foods) (Mayén et al., 2014; Allen *et al.*, 2017). School nutrition policies that restrict the availability of unhealthy foods have been proposed as an equitable obesity prevention intervention, given their reduced reliance on individual-level behaviour change (Backholer *et al.*, 2014). Understanding potential differences in policy implementation by SEP is therefore important for assessing equity of policy impact. Finally, neither of the previous reviews reported barriers or enablers to policy implementation; knowledge of these factors would aid understanding of the feasibility of such policies and contribute to planning the effective implementation of such policies.

The aim of this review was to synthesize the international literature on the impact of implemented primary school nutrition policies on the healthiness of foods available in schools. A secondary objective was to report on the impact of the included policies on the availability of food in school in relation to SEP.

## METHODS

This review was conducted in line with the Preferred Reporting Items for Systematic Reviews and Meta-Analyses (PRISMA).

### Search strategy

Keyword and subject heading searches related to nutrition policy and schools were conducted in seven databases (Ovid MEDLINE, Embase, Cochrane Library, CINAHL Plus, ERIC, Informit Health Collection and Informit A+ Education) on 30 March 2015 and updated on 9 June 2021. No limits were placed on country or publication date. Searches were limited to the English language. An example of the search strategy is provided in Appendix A.

### Eligibility criteria

The setting was limited to primary schools. Studies that reported only combined results for primary and secondary schools were excluded. A school nutrition policy was defined as a formally adopted policy that provides a guide for food- and nutrition-related activities within a school. As the review objectives concerned factors influencing policy implementation, only policies that had been implemented were included. Studies that measured the difference in the proportion or absolute amounts of food available as an outcome were included. Study designs eligible for inclusion were pre-and-post studies (including repeat cross-sectional studies), with or without a comparison group, and post-only studies that compared food availability in schools with the policy to schools without it.

### Selection of studies for inclusion

The screening and data extraction was carried out by several of the authors (L.G-D., R.B., T.B-R., M.B. and C.P.). All the authors were public health researchers. At each stage of the screening and extraction, each study retrieved from the search was allocated two authors to review it. Titles and abstracts were screened for relevance by two authors. For papers deemed potentially relevant, the full text article was assessed against the eligibility criteria (Table 1) by two authors; full texts meeting all criteria were included in the review. Where multiple articles reported on the same study, we used the article with more comprehensive and up-to-date data.

**Table 1:** Eligibility criteria for studies for inclusion in this systematic review

Inclusion criteria	Exclusion criteria
• English language • Published in a peer-reviewed journal • Setting: primary school • Intervention: implemented school nutrition policy • Outcomes: availability of foods and beverages • Study design: pre-and-post studies (with or without a comparison group and including repeat cross-sectional study designs), post-only studies which compare availability in schools with policy of interest and schools without policy of interest	• Intervention: policy which focused on undernutrition, hunger, specific micronutrient deficiency or employee health; policy adopted for the purposes of a research trial • Report only combined results for primary and secondary schools

### Data extraction

Data were extracted from eligible articles independently by two authors, with discrepancies discussed and resolved among four authors. Data extracted included: author/s, year of publication, aim, study design, year/s of data collection, study location, response rate and sample size, policy description (policy aim, an overview of policy content and the date of introduction or expected implementation), policy level (the highest level of governance at which the policy had been adopted, e.g. school, district, state/provincial, national) and requirements for policy compliance (mandatory or voluntary), data collection method, statistical analysis methods, results related to review objectives (e.g. changes to the availability of foods, overall and by an indicator of SEP), and reported barriers or enablers to policy implementation.

### Quality assessment

The risk of bias in individual studies was assessed using a modified version of the Effective Public Health Practice Project Quality Assessment Tool for Quantitative Studies (Effective Public Health Practice Project, 2010). Studies were assessed against criteria related to selection bias, study design, confounders, data collection methods, and withdrawals and drop-outs, and given a rating of ‘strong’, ‘moderate’ or ‘weak’ for each criterion. As studies were observations of ‘real world’ policies, blinding would not be possible and was therefore excluded from the quality assessments. The modified version of the tool has previously been used for this reason (Beauchamp *et al.*, 2014; Boelsen-Robinson *et al.*, 2015).

### Data synthesis

Due to the heterogeneity of policies and type of outcome between studies, the findings were synthesized narratively. Results were synthesized into three sections relevant to the aims of the study: the impact of school nutrition policies on the availability of food and beverages in school, the impact in relation to SEP and the barriers and enablers to the implementation of policies. Summaries of the characteristics of the included studies are presented in a table and include information on the author, study design, participants (number of schools and country), policy (policy level, requirement for compliance, policy description and date introduced), and outcomes (results, the impact of policy in relation to SEP, reported barriers and enablers).

## RESULTS

### Characteristics of the included studies

**Fig. 1:**
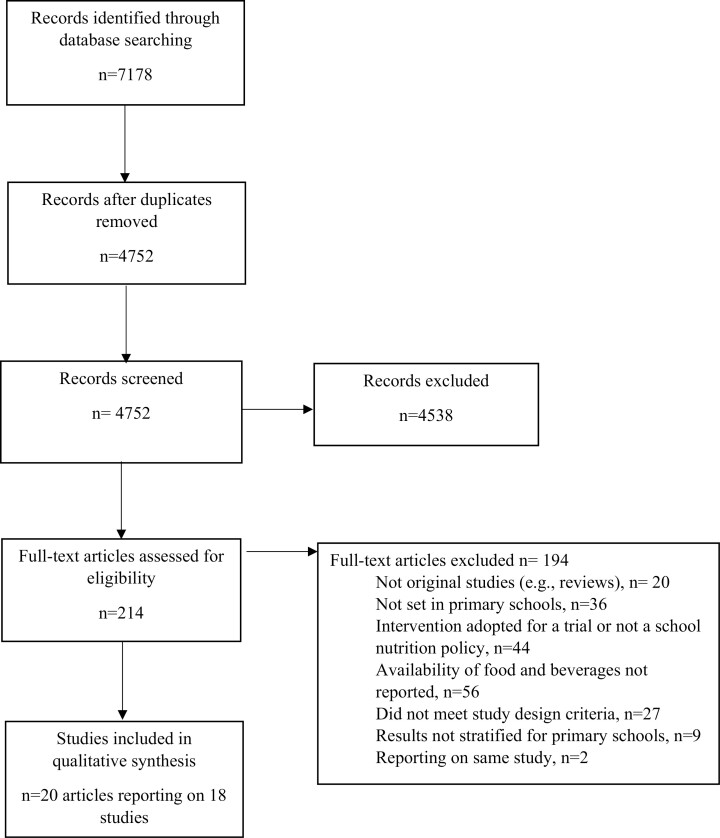
Selection process for studies included in this systematic review.

A total of 7178 records were identified through database searching. After removing duplicate references and screening titles and abstracts, 214 records were deemed relevant for full-text review. Twenty articles (reporting on eighteen studies) met the inclusion/exclusion criteria and were included for data extraction and synthesis (Figure 1). Most studies were based in the USA (*n* = 14), with the remaining studies conducted in the United Kingdom (UK; *n* = 1), Sweden (*n* = 1), Brazil (*n* = 1) and Mexico (*n* = 1) (Table 2). Combined, the studies included over six thousand primary schools. Fourteen studies used a pre-and-post study design whilst four used a post-only study design (Table 2). There was variability between studies, however, the majority of studies collected post-policy measurements within one to two years after policy implementation (Table 2). Based on the modified Effective Public Health Practice Project Quality Assessment Tool for Quantitative Studies, twelve studies were rated as weak quality and six as moderate quality (Table 2; Appendix B), suggesting the risk of bias in the included studies to be moderate to high.

**Table 2:** Characteristics and results of studies included in this systematic review

Authors (year)	Study design	Participants	Policy				Data collection	Outcomes			Quality rating
			Level	Requirement for compliance	Policy description	Policy introduction	Data collection date	Overall result	Impact in relation to SEP^a^	Barriers and enablers	
Behrens *et al.* (2018)	Pre- and post-study, no comparison group	Schools in Southern Colorado, USA Kindergarten to fifth grade 2009: 15 schools 2015: 15 schools Study schools were from a high-need school district	District	Unclear	Districtwide school food preparation best practices. Unhealthy options reduced or removed; healthier options made available. Policy implementation facilitated by training for cafeteria employees.	Phased in with full implementation by May 2015	Pre-implementation: Jan 2009–May2010 Intervention phases Phase 2: Aug 2010-May 2011 Phase 3: Aug 2011-Dec 2012Phase 4: January 2013–May 2015	Increased offerings of: • raw/steamed vegetables and fresh fruit (*p* < 0.01) • fresh/low-sodium potato sides (p < 0.01) Decreased offerings of: • fried/high sodium potato sides (p < 0.01) • bread removed No change in offerings of: • dessert	Not reported	Not reported	Weak
Belansky *et al.* (2013), with additional details from Belansky *et al.* (2010), as indicated	Pre- and post-study, no comparison group	Elementary schools in rural Colorado, USA 2005: 32 schools 2011: 40 schools Study schools were rural low-income schools	District	In 2007, most policies used language which recommended rather than mandated compliance (Belansky *et al.*, 2010)	A federal mandate required school districts to create Local Wellness Policies. In 2007 most included nutrition guidelines and regulations for vending machines, school stores and à la carte service while fewer placed limits on nutrients (Belansky *et al.*, 2010).	School districts were required to create policies by June 2006	Pre-implementation: 2005 Post-implementation: 2011	No significant changes to: • number of fruit lunch choices or number of vegetable lunch choices • % of schools with à la carte menu offering fruits and vegetables, % of schools with à la carte menu offering candy, high-fat snacks, or high-calorie fast foods, % of schools with vending machines with carbonated beverages, or % of schools with vending machines with high-fat, high-calorie items	Not reported	In 2007, foodservice managers reported that a lack of financial resources was a barrier to purchasing a wider selection of healthy foods (Belansky *et al.*, 2010)	Weak
Boehm *et al.* (2020)	Post-only study, comparison group	154 elementary schools in New York State, USA The districts were identified as high need based on a community needs index that placed them below the statewide median in indicators of poverty, educational attainment, and childhood obesity	School district	Unclear. Implementation status assessed between March 2016 and July 2018	Various, including policies on competitive foods.	Unclear	Policy strength assessed between February 2015 and September 2017	No significant association between the strength of the policy (strong, weak, none) and the implementation status for: • vending machines/stores/concession complying with Smart Snacks • beverages sold during school complying with Smart Snacks • food served during celebrations having restrictions • fundraisers selling foods having restrictions	Not reported	Not reported. In discussion, authors use the Ambiguity-Conflict Model of Policy Implementation which suggests the amount of conflict and ambiguity impact the implementation of a policy. Applying this model to understand the lack of difference, the authors suggest that due to low ambiguity and conflict, there was high implementation of nutrition standards for competitive foods, regardless of policy strength.	Weak
Chriqui *et al.* (2013)	Post-only study, comparison group	Elementary schools in USA 2008–09 through 2010–11: Pooled sample of 1,919 respondent schools over 3 years (1,582 unique schools)	School district, state	Mandatory	Various school district and state policies, including limits on (i) sugar, (ii) fats and (iii) sodium in foods and bans on (iv) candy, (v) sugar-sweetened beverages (soda, sports drinks, and other sweetened fruit drinks not 100% juice), (vi) regular soda, and (vii) high-fat (2% or whole) milk	Various (multiple policies included)	2008–2009 through 2010–2011	Schools covered only by school district limits/bans (compared with schools not covered by school district or state limits/bans): • no significant difference in odds of availability of sweets, candy, regular-fat baked goods and salty snacks • significantly less likely to have regular-fat ice cream (OR^b^ 0.5, 95% CI^c^ 0.2–0.9), sugar-sweetened beverages (OR 0.2, 95% CI 0.7–0.9) and high-fat milk (OR 0.4, 95% CI 0.2–0.8) available Schools covered only by state limits/bans (compared with schools not covered by school district or state limits/bans): • no significant difference in odds of availability of sweets, candy, salty snacks, sugar-sweetened beverages and regular soda • significantly less likely to have regular-fat baked goods (OR 0.5, 95% CI 0.3–0.9), regular-fat ice cream (OR 0.5, 95% CI 0.2–0.9) and high-fat milk (OR 0.4, 95% CI 0.2–0.7) available Schools covered by both school district and state limits/bans (compared with schools not covered by school district or state limits/bans): • no significant difference in odds of availability of candy, salty snacks, sugar-sweetened beverages, regular soda and high-fat milk • significantly less likely to have sweets (OR 0.6, 95% CI 0.4–0.8), regular-fat baked goods (OR 0.4, 95% CI 0.2–0.6) and regular-fat ice cream (OR 0.4, 95% CI 0.3–0.6) available	Low-SEP schools (indicated by the by the percentage of students eligible for reduced-price or free lunch) more likely to sell sugar-sweetened beverages when their sale is banned by state law than mid- or high-SEP schools (not significant)	Not reported	Moderate
Cluss *et al.* (2014)	Pre- and post-study, no comparison group	Elementary schools in Pennsylvania, USA 2005: 7 schools 2011: 7 schools Students at study schools were predominantly from Caucasian and low-income backgrounds	School district	Unclear	Changes to the school lunch program to reduce amounts of total fat, saturated fat and trans fat	Various changes made from the 2005–06 school year through to the 2011–12 school year	Data collected annually for the 7 years	Decrease in: • % of entrees offered on the menu which were Whoa foods (foods that should only be eaten once in a while or for special treats) from 30% in 2005 to 0% in 2011 • % of all foods offered on the menu which were Whoa foods from 22% in 2005 to 0% in 2011	Not reported	Not reported	Moderate
Cummings *et al.* (2014)	Pre- and post-study, no comparison group	Elementary schools in Los Angeles County, California, USA 2010–11: 931 schools 2011–12: 947 schools	School district	Unclear	Incorporation of Institute of Medicine recommendations in menu planning	Changes made for the 2011–12 school year menus	Pre- implementation: October 2010 Post- implementation: October 2011	Breakfast: • significant decrease in energy, protein, fibre, total fat, saturated fat, sugar and sodium content Lunch: • significant increase in protein and sodium content. Significant decrease in fibre content. No significant change in energy, total fat, saturated fat and sugar content	Not reported	Not reported	Weak
Haroun *et al.* (2011), with additional details from Pearce *et al.* (2013), as indicated	Pre- and post-study, no comparison group	Primary schools in England, UK 2005: 151 primary schools^d^ 2009: 136 primary schools	National	Mandatory	Food-based standards (to increase access to healthier foods and limit availability of less healthy foods) and nutrition-based standards (to ensure food contains appropriate amounts of energy, iron, fat, sugar and salt)	September 2008	Pre- implementation: 2005 Post-implementation: 2009	Compared with 2005, in 2009: • schools provided significantly more vegetables and salad; fruit; starchy foods not cooked in fat; milk, yoghurt and milky drinks; water; fruit juice; and fruit-based desserts • schools provided significantly fewer desserts not containing fruit; condiments; starchy foods cooked in fat; and non-permitted items such as savoury snacks and confectionery • there was no significant difference in provision of main dishes or baked beans • there was a significant change in portion size of 13% of comparable foods available (Pearce *et al.*, 2013)	Not reported	Not reported	Weak
Ishdorj *et al.* (2016)	Pre- and post-study, no comparison group	Elementary schools in central Texas, USA April/May 2012: 3 schools Oct/Nov 2012: 3 schools Schools varied in the proportion of students eligible for reduced-price/free lunches (mean: 66%; range: 31–99%).	National	Mandatory	The nutrition standards of The National School Lunch Program were updated to align with the most recent Dietary Guidelines for Americans.	June 2012	Pre-policy: April and May 2012 Post-policy: October and November 2012	Significant changes in: Nutrient density score (mean % of daily values/100g): • decrease for red/orange vegetables, beans and peas, and French fries (*p* < 0.05) • increase for potato wedges (*p* < 0.05) Energy density (kilocalories/100g): • ncrease for dark green and non-starchy vegetables • decrease for starchy vegetables Nutrient density per dollar: • decrease for beans and peas, potato wedges, French fries, and mashed potato • increase for “other starchy” vegetables % plate waste: • increase overall	Not reported	Not reported	Moderate
Jimenez-Aguilar *et al.* (2017)	Pre- and post-study, no comparison group	Public elementary schools in Mexico N = 39	National	Mandatory	The General Guidelines for Dispensing or Distribution of Foods and Beverages at School Food Establishments aimed to ensure that schools dispense healthy foods and beverages with low energy density, prepare them hygienically, and promote healthy habits.	Implementation began in January 2011, Phased in with full implementation by 2013	2011–2012 and 2012–2013	Significant changes in: Availability: • increase in average portion size of SSB^e^ (*p*>0.01), plain bottled water (*p* = 0.04) • increase in availability in average portions of cookies, snack cakes, and desserts (all *p* = 0.01) • decrease in the average portions of fruits and vegetables (*p* = 0.02) and decrease in plain bottled water (*p* = 0.06) Compliance: • decrease in compliance with total fat (*p* = 0.02), and sodium in cookies, snack cakes, and desserts (*p* = 0.03)	Not reported	Not reported. In discussion, authors suggest the poor compliance observed may reflect the lack of penalty for non-compliance	Weak
Kubik *et al.* (2010)	Post-only study, comparison group	Elementary schools in USA 2006: 214 schools	School district, state	Varied (some voluntary, some mandatory)	Various policies that recommend or require prohibition of offering of junk foods in school stores and vending machines	Various (multiple policies included)	Jan-Oct 2006	Schools covered by state policies that require prohibition: • significantly less likely to offer junk food, compared with schools covered by state policies that neither require nor recommend prohibition • no significant difference in % of schools offering junk food, compared with schools covered by state policies that recommend prohibition Schools covered by state policies that recommend prohibition: • no significant difference in % of schools offering junk food, compared with schools covered by state policies that neither require nor recommend prohibition Schools covered by school district policies that require prohibition: • no significant difference in % of schools offering junk food, compared with schools covered by school district policies that neither require nor recommend prohibition • no significant difference in % of schools offering junk food, compared with schools covered by school district policies that recommend prohibition Schools covered by school district policies that recommend prohibition: • no significant difference in % of schools offering junk food, compared with schools covered by school district policies that neither require nor recommend prohibition	Not reported	Not reported	Weak
Long *et al.* (2010)	Pre- and post-study, comparison group	School districts in Connecticut, USA 2006: 151 school districts 2007: 104 school districts	State	Voluntary	School districts that comply with limits on fat, sugar and portion sizes receive additional funding	Launched in the 2006–07 school year	2005–2006 (Baseline) to 2006–2007	Significantly greater reduction in the number of unhealthy à la carte snack categories offered from 2006 to 2007 in elementary schools in school districts that chose to comply with the limits compared with elementary schools in school districts that did not	No significant effects of SEP on adoption of the policy or change in availability of unhealthy à la carte snacks	Not reported	Weak
Ohri-Vachaspati *et al.* (2012)	Post-only study, comparison group	Elementary schools in USA 2010: 620 schools	Federal	Voluntary	Program providing reimbursement to schools with low-income students for offering fresh fruits and vegetables outside meal times	Expanded funding for the program mandated in 2008 (the program started as a pilot in 2002)	February to June of the 2009–2010	Schools participating in the program were significantly more likely to offer fresh fruit in lunch meals than schools not participating in the program. No significant difference in the odds of offering vegetables (excluding potatoes) or salad between schools participating in the program and those not	Not reported	Not reported	Weak
Ohri-Vachaspati *et al.* (2016)	Pre- and post-study, no comparison group	Elementary schools in USA 2006–07: 520 schools 2012–13: 546	National	Mandatory	Schools participating in the National School Lunch Program must include both a fruit and vegetable each day, and a variety of vegetables must be offered on a weekly basis	July 2012	Pre-policy: 2006–2007 Post-policy: annually until 2013	Percentage of schools offering a salad bar significantly increased over time (p for trend <0.001)	Adjustments for SEP are made in the analysis. No stratification of results by SEP	School-level resources and programs associated with the presence of a salad bar: • significant predictors included participation in Fresh Fruit and Vegetable Program, participation in Team Nutrition, participation in Farm to School Program, and having school lunch provided by foodservice management company • for every additional resource/program, the odds of having a salad bar increased • Non-significant predictors were having a full-service kitchen, a dietitian/ nutritionist on staff, a garden that students participate in, providing nutrition education to students, or having school lunch provided by school system food service	Moderate
Patterson *et al.* (2015)	Pre- and post-study, no comparison group	Primary schools in Sweden 2011: 191 schools 2013: 97 schools	National	Mandatory	Lunches should be based on Swedish nutritional recommendations: 30% of daily energy from lunch; suggested serving frequencies for certain foods; guidelines on how to evaluate the nutritional adequacy of the menu; and how to make the school meal an integral part of the school day	July 2011	Pre-policy: Spring 2011 Post-policy: spring 2013	Food provision/choice: • proportion of schools offering a vegetarian dish significantly increased • no change in choice of main dish, or salad buffet Adherence to serving guidelines: • significantly more adherence to serving guidelines for skimmed milk (daily) and fish (min 4 times/4weeks) (*p* < 0.05) • no differences in salt, SSB, desserts, fatty fish, sausages, low fat margarine, or blood pudding Nutritional adequacy: • significantly more adherence to nutrient recommendations for fibre and iron • no difference in fat quality or vitamin D Availability of other foods/drinks: • no difference in the availability of vending machine, cafeteria, or water	Not reported	Not reported. In the discussion, the authors comment that the ambiguity as to what was expected of schools and what the consequences of non-compliance would be was likely a barrier. They suggest regular monitoring would enable improvements in school meal quality over time	Weak
Phillips *et al.* (2010)	Pre- and post-study, no comparison group	Elementary schools in Arkansas, USA 2004: 416 schools 2008: 433 schools	State and district	Some aspects mandatory (restriction of vending machine access), other aspects unclear	State act that created a state-wide committee to develop nutrition policy recommendations, restricted access to vending machines during the school day, and required school districts to establish committees to develop local policies	The act was passed in 2003	Baseline and Year 5	Significant decrease in: • % of schools offering whole white milk and whole chocolate milk in cafeteria Significant increase in: • % of schools offering low fat chocolate milk, skim white milk and skim chocolate milk No significant change in: • % of schools offering low fat white milk in cafeteria • % of schools with beverage vending machines with sodas, fruit drinks, 100% fruit juices and water • % of schools with snack food vending machines with chocolate candy, other candy, cookies, chips, low-fat low-sugar cookies, low-fat crackers and low-fat chips Decrease (significance not reported) in: • % of schools with snack food vending machines with ice cream	Not reported	Not reported	Weak
Samuels *et al.* (2010)	Pre- and post-study, no comparison group	Elementary schools in low-income communities in California, USA 2005: 6 schools 2008: 6 schools Schools were located in low-income communities	State	Mandatory	State legislative standards that limit the types of foods and beverages elementary schools can sell. The standards include some nutrient limits	Passed in 2005, with full implementation of the food standards required by 2007 and of the beverage standards by 2009	2005 and 2008	Proportion of foods adherent to the standards increased from 0% in 2005 to 61% in 2008. Proportion of drinks adherent to the standards increased from 57% in 2005 to 100% in 2008.	Not reported	Not reported	Moderate
Soares *et al.* (2017)	Pre- and post-study, no comparison group	School meals purchased in the municipality of Santa Catarina, Brazil. ~50 public schools for infant and primary education and 5700 students (>4000 rural)	National	Mandatory	The National School Feeding Program (NSFP) guidelines were modified to promote healthy eating at school and local family farm production. Regulations included criteria for food procurement. Purchasing products high in sodium, sugar, saturated- or trans-fats was restricted. Low nutrition drinks were prohibited. A minimum of 3 portions of fruit and vegetables was recommended to be included weekly in school menus. The provision of the NSFP with products purchased directly from local family farmers, prioritizing organic production and the most vulnerable producers was mandated.	2010	Pre-policy: 2008 and 2009 Post-policy: 2010 and 2011	Change in proportion of daily quantities (kg/day) of foods purchased: • significant increase in recommended foods (*p* = 0.005) • significant decrease in controlled (unhealthy) foods (*p* = 0.005) • significant decrease in fruit (*p* = 0.03) • significant increase in legumes and vegetables (*p* < 0.05) • significant decrease in foods high in sugar (*p* = 0.02) • no change in concentrated products (e.g. biscuit mixes), meat, cheese and sauces with high sodium and/or saturated fat Change in food variety (number of different food items included in the purchase list each year): • 10 new recommended (healthy) food products included, 1 removed • 2 controlled (unhealthy) products removed	Not reported	Not reported	Weak
Taber *et al.* (2015)	Pre- and post-study, comparison groups	40 states in the USA 2004: 1410 public schools grade 5. 2007: 1430 public schools grade 8	State	Varied	Different depending on law and state but focused on competitive food laws	Varied depending on law and state (based on laws that were in place as of 31st December of the year)	Spring 2004 and spring 2007	The association between the strength of the state’s law (strong, weak, none) and the school food environment (measured using the Healthy School Food Environment Index [HSFEI], Healthy School Beverage Environment Index [HSBEI], and Healthy School Overall Environment Index [HSOEI]. A higher score represents a healthier environment): • there was no association between strong laws and index scores in 5th grade (overall or by school SEP) • strong laws were associated with higher HSFEI and HSOEI scores in 8th grade (regardless of school SEP) • there was no association between states with weak laws and the school food environment	• Schools were classified into SEP tertiles. • The distribution of law category (none, weak, strong) was similar across SEP tertiles in both grades. • Strong laws were positively associated with HFSEI in grade 8 regardless of SEP • Competitive beverage laws more strongly associated with HSBEI in low-SEP vs medium-or high-SEP schools in grade 8 • High-SEP schools sold more healthy items than low-SEP schools regardless of state laws	Not reported	Moderate

Socioeconomic position.

Odds ratio.

Confidence intervals.

Source: Nelson *et al.* (2006).

Sugar-sweetened beverages.

The types of policies vary. Broadly, they included policies that intended to limit the availability of various unhealthy foods, beverages and/or nutrients in schools (*n* = 7), increase the offerings of fruits and vegetables (*n* = 2), reduce the availability of unhealthy foods *and* increase the availability of healthy foods (*n* = 2), or incorporate nutritional recommendations or best practice guidelines into school food outlets (*n* = 5). Two studies rated the strength (strong, weak, none) of policies. The strength of a policy was based on factors such as the comprehensiveness of the policy and the specificity of its language (Table 2).

### Impact of school nutrition policies on the availability of food and beverages in school

Of the 18 studies, 13 reported some positive impacts on food availability and no negative impacts (Table 2). Three studies reported an increase in the availability of healthy foods (e.g. salad bars) (Ohri-Vachaspati et al., 2012, 2016; Patterson *et al.*, 2015), five reported a reduction in unhealthy foods available (Kubik *et al.*, 2010; Long *et al.*, 2010; Samuels *et al.*, 2010; Chriqui *et al.*, 2013; Cluss *et al.*, 2014), and five of the studies found both an increase in healthy foods and decrease in unhealthy foods available in schools (Phillips *et al.*, 2010; Haroun *et al.*, 2011; Pearce *et al.*, 2013; Taber *et al.*, 2015; Soares *et al.*, 2017; Behrens *et al.*, 2018). Unhealthy food definitions ranged from specific nutrients (e.g. saturated fats, sugar, and sodium) to food categories (e.g. sugar-sweetened beverages). The change in availability occurred in cafeterias, school stores and/or vending machines.

Two studies (assessments of district-level Local Wellness Policies in Colorado and New York State, USA) reported no changes in food availability (Belansky et al., 2010, 2013; Boehm *et al.*, 2020).

In two studies, policies resulted in a mix of positive and negative outcomes. After the introduction of district-level policies in Los Angeles County, California, USA there were significant decreases in the energy, protein, fibre, total fat, saturated fat, sugar and sodium content of breakfasts, and significant increases in the protein and sodium content, a significant decrease in fibre content and no significant changes in the energy, total fat, saturated fat or sugar contents of lunches (Cummings *et al.*, 2014). In central Texas, the USA, updates to the nutrition standards of The National School Lunch Program resulted in a significant decrease in nutrient density (mean % of daily values/100g) of French fries and energy density (kcal/100 g) of starchy vegetables, and significant increases in the energy density of dark green and non-starchy vegetables. However, there was a decrease in the nutrient density of red/orange vegetables, beans and peas (Ishdorj et al., 2016).

One study reported only negative impacts on the healthiness of food availability. Jimenez-Aguilar *et al.* (Jimenez-Aguilar *et al.*, 2017) assessed compliance with The general guidelines for dispensing or distribution of foods and beverages at school food establishments in Mexico over two academic years. They found poor compliance and a significant increase in the availability of less healthy foods and a decrease in healthier options over time. The authors suggest this may be attributable to the lack of consequences for non-compliance.

### Impact of school nutrition policies on the availability of foods and beverages in school according to an indicator of socioeconomic position

Of the eighteen studies, eight reported the impact of policies on food availability by SEP. Five of these were based exclusively in schools classified as low SEP (Samuels *et al.*, 2010; Belansky *et al.*, 2013; Cluss *et al.*, 2014; Behrens *et al.*, 2018; Boehm *et al.*, 2020) with three out of the five reporting positive impacts on food availability and two reporting no impact.

Three of the studies compared the findings by SEP. One cross-sectional study compared the availability of sugar-sweetened beverages sold in schools when their sale was banned by state law in low-, mid- and high-SEP schools (indicated by the percentage of students eligible for reduced-price or free lunch) (Chriqui *et al.*, 2013). Sugar-sweetened beverages were sold in 25% of low-SEP schools with a state policy banning their sale compared with 10% of mid-SEP schools and 5% of high-SEP schools. Soda was sold in 3% of low-SEP schools with a state policy banning their sale compared with 2% of mid-SEP schools and 1% of high-SEP schools. However, due to the cross-sectional nature of the study design, it is not possible to ascertain the degree to which sales changed over time between the different SEPs.

Another study classified districts into SEP tertiles (high, middle and low need districts) based on a composite district-level variable (District Reference Group) (Long *et al.*, 2010). Overall, there was a significant reduction in the number of unhealthy à la carte snack categories offered in school districts that chose to adopt the policy compared with elementary schools in school districts that did not. However, there were no significant effects of SEP on policy adoption or change in the availability of unhealthy à la carte snacks.

One study assessed the association between the strength of state competitive food (foods and beverages sold in a school outside of the school meal programs) laws in 40 states in America and the foods available for sale in schools (as a measure of the healthiness of the school food environment, beverages environment and overall) at two-time points (2004 and 2007) (Taber *et al.*, 2015). Schools were classified as high-, medium- or low-SEP, based on the median household income of the student’s postcode. There was an association between states with strong competitive food laws and healthy school food environments in 2007, regardless of SEP. Some SEP differences were observed in 2007, with high-SEP schools rated as healthier food and beverage environments overall relative to low-SEP schools, regardless of state laws. This difference was due to the disparity in healthy (as opposed to unhealthy) items available. Conversely, competitive beverage laws were more strongly associated with healthier beverage environments in low-SEP compared to medium-or high-SEP schools.

### Barriers and enablers to the implementation of policies

Two studies reported barriers or enablers to policy implementation. In the evaluation of district-level Local Wellness Policies in Colorado, USA, in which no changes to school food availability were identified, food service managers reported a lack of financial resources as a barrier to offering a wider selection of healthy foods (Belansky *et al.*, 2010). Another study assessed the percentage of schools offering a salad bar before and after updates were made to the National School Lunch Program (Ohri-Vachaspati *et al.*, 2016). The authors used multivariable logistic regressions to analyze school-level resources (resources included the availability of a dietitian/nutritionist on staff, a full-service kitchen, school garden and nutrition education provided to students) and programs (programs included the Fresh Fruit and Vegetable Program, Team Nutrition Program and Farm to School Program) associated with the presence of a salad bar. The study identified several significant predictors, including participation in the programs and having school lunch provided by a food service management company. For every additional resource/program, the odds of having a salad bar increased by 21%.

## DISCUSSION

The results from this review demonstrate that implemented school nutrition policies were mostly associated with greater availability of healthier foods and/or lower availability of less healthy foods. Furthermore, the findings from this review indicate that school nutrition policies are likely to be an equitable obesity prevention intervention.

The finding that school nutrition policies are generally associated with positive impacts on the availability of foods in schools confirms the findings of two previous systematic reviews, although the policy definitions and eligibility criteria differ. Jaime and Lock’s review of school nutrition policies in primary and secondary schools across the world included four studies reporting on food availability as an outcome, with the studies predominantly focusing on the availability of fruits and vegetables offered at school lunch. All four reported increased fruit and vegetable availability after the policy introduction (Jaime and Lock, 2009). These policies were, however, adopted for the purposes of research trials, which may have artificially increased the degree of policy implementation—our review adds to this evidence that ‘real world’ implemented school nutrition policies are effective. In addition, our review indicates that policies may increase the availability of a range of healthy foods and reduce the availability of unhealthy foods (e.g. desserts and unhealthy snacks). Chriqui *et al.* reviewed school nutrition policies adopted in the USA and found that policies were associated with changes to food availability in the expected healthy direction in five of seven studies reporting on this outcome (the remaining two produced mixed results) (Chriqui *et al.*, 2014). Our review confirms Chriqui’s findings and adds to these by showing similar findings in other high-income countries.

Further to the two previous reviews, our review also aimed to understand the potential equity impacts of school nutrition policies on the availability of foods in primary schools. The results of this review suggest that school nutrition policies are likely to have a positive impact on more disadvantaged schools, with three of five studies reporting positive impacts in schools classified as low-SEP and a further three studies reporting no difference in impact between schools classified as higher or lower SEP. These results support the hypothesis that well-implemented school nutrition policies that restrict the sale of less healthy foods are unlikely to exacerbate the socioeconomic gradient of poor nutrition (Backholer *et al.*, 2014). In our review, two studies set in low-income communities reported no significant association of policies on foods available in school (Belansky *et al.*, 2013; Boehm *et al.*, 2020). The schools in the study by Belansky *et al.* (Belansky *et al.*, 2013) were located in rural areas, which may have posed specific implementation challenges. For example, other studies have reported that rural schools have difficulty accessing healthier foods because of their rural location (Downs *et al.*, 2012). Overall, the number of studies that reported results by SEP limits drawing strong conclusions in relation to equity impacts. Greater reporting of disaggregated results by SEP and rurality/remoteness is needed to determine whether there are differences in the implementation of school nutrition policies and, if so, to understand factors that may contribute to this.

Two studies in this review reported on the barriers or enablers to the implementation of the studied school nutrition policies. Additional resources and programs were found to increase the likelihood of a school having a salad bar (Ohri-Vachaspati *et al.*, 2016) and lack of financial resources was reported as a barrier to purchasing a wider selection of healthy foods (Belansky *et al.*, 2010). Financial barriers (e.g. higher costs of purchasing healthier foods and reduced profit and revenue from selling healthier options) were also identified as key deterrents to school nutrition policy implementation and compliance in a recent systematic review of barriers and enablers to implementing healthy food policies in schools (Ronto *et al.*, 2020). Other barriers reported in that review included difficulty accessing foods that comply with policies, and easy access to unhealthy food outlets surrounding schools, while enablers included adequate funding, and clear, well-communicated policies (Ronto *et al.*, 2020). Further to this, a recent systematic review on the business outcomes of healthy food service initiatives found that favourable business outcomes were achieved in certain school settings (canteens/cafeterias/tuckshops) but not in others (vending machines), suggesting financial support from governments could enable policy implementation and compliance (Thorpe *et al.*, 2021).

Given the finding that implemented school nutrition policies generally have a positive impact on the availability of healthy foods in primary schools, an important follow-up to this review is to evaluate the impact of school nutrition policies on diet quality and anthropometric measures. It has been suggested students may compensate for restricted foods by purchasing other less healthy items which are still available or by bringing such items from home (Hawkes *et al.*, 2015). A study of a school nutrition policy adopted in a school district in Texas, USA found that the mean daily consumption of candy and snack chips did not change after policy introduction, with students compensating for banning these items in snack bars by purchasing them from vending machines, where they were not banned (Cullen *et al.*, 2006). The type of policy which is implemented (e.g. partial or full restriction of less healthy items) and whether the policy is supported by other strategies are likely to be important factors in the impact that policies have on consumption outcomes (Hawkes *et al.*, 2015). While previous reviews of school nutrition policies (Jaime and Lock, 2009; Chriqui *et al.*, 2014; Micha *et al.*, 2018) included consumption and adiposity outcomes, these reviews covered a limited population, included policies adopted for the purposes of trials, or did not identify barriers and enablers. More studies in this area are warranted to determine the impact of real-world school nutrition policies on consumption and adiposity outcomes, the characteristics of policies that are most effective and whether there are differences in impact by SEP.

### Strengths and limitations

Our review was conducted in line with the preferred reporting items for systematic reviews and meta-analyses (PRISMA), which aims to improve the reporting of systematic reviews. Screening of potentially relevant full text articles, data extraction and quality assessment were all conducted by at least two authors. The inclusion of only school nutrition policies that have been implemented by policymakers and practitioners, rather than researchers, increases external validity and the synthesis of potential equity impacts is novel.

Due to restricting peer-reviewed literature there may be additional policies implemented of relevance in the grey literature. Future research could work towards robust evaluations of these policies. The majority of included studies were assessed as weak quality, with a major contributor to this being study design. The majority of pre-and-post studies in this review did not employ a comparison group. In the one pre-and-post study that included a comparison group, positive changes to the foods available in school were found in the comparison group, although not to the extent found in the group that adopted a policy (Long *et al.*, 2010). This demonstrates that wider social and cultural changes need to be considered when interpreting the results of the other included pre-and-post studies. While multiple pre-and-post studies were classified as repeat cross-sectional studies, in some of these studies, a substantial proportion of the schools participating at both time points were the same. The remaining studies included in this systematic review employed a post-only (i.e. cross-sectional) study design, which limits the extent to which the differences in school food environment can be attributed to the presence of a school nutrition policy. A second major contributor to the weak quality of included studies was data collection; many studies used school staff-completed surveys that had not been shown to be valid or reliable and may have been susceptible to social desirability bias. The quality of the evidence included in this review indicates that, where possible, future school nutrition policy evaluations should include a comparison group so that the extent of change to the school food environment that is due to policies, and the extent that is explained by wider social and cultural changes, can be determined. The use of objective or validated measures of food availability would further improve the quality of evaluations.

The studies identified in this review were predominantly based in the USA (*n* = 14) and in either high-income (*n* = 16) or upper middle-income countries (*n* = 2), potentially limiting the generalizability of the findings given education systems and school food services differ between countries.

### Implications

School nutrition policies are generally associated with greater availability of healthier items and/or lower availability of less healthy items, which demonstrates the feasibility of sustained and effective policy implementation. Given the growing global burden of disease attributable to poor dietary habits and the opportunity schools provide to influence dietary habits for all children, school nutrition policies represent a feasible and promising mechanism for improving diet quality. However, the barriers reported in this review and the wider literature highlight that there are many factors that contribute to how successfully policies are implemented. To ensure optimal implementation of school nutrition policies, consideration of these factors during policy development is needed.

## CONCLUSION

Our review has found that primary school nutrition policies are generally associated with greater availability of healthier foods and/or lesser availability of less healthy foods. Based on the limited number of studies reporting results by SEP, these policies also appear to be effective for schools classified as higher and lower SEP. Combined with the broader literature, school nutrition policies offer a feasible and promising intervention to improve diet quality. Further research that reviews the impact of policies on consumption and anthropometric outcomes is needed and should include an analysis of the impact of SEP.

## Supplementary Material

daac084_suppl_Supplementary_Appendix_A

daac084_suppl_Supplementary_Appendix_B
