# Supplementary material for: The impact of primary school nutrition policy on the school food environment: a systematic review
Source: Health Promot Int. 2022 Sep 27;37(5):daac084. doi: 10.1093/heapro/daac084 (PMC9514228; doi:10.1093/heapro/daac084)
Supplement: daac084_suppl_Supplementary_Appendix_A [file daac084_suppl_supplementary_appendix_a.docx]

**Appendix A:**

*Example of search strategy for Embase database*

| **Hedge** | **Search terms** |
| --- | --- |
| **Food** | "wellness polic*"  nutrition* NEAR/3 polic*  nutrition* NEAR/3 standard*  nutrition* NEAR/3 guide*  diet* NEAR/3 polic*  diet* NEAR/3 guide*  food* NEAR/3 polic*  food* NEAR/3 guide*  beverage* NEAR/3 polic*  beverage* NEAR/3 guide*  canteen* NEAR/3 polic*  canteen* NEAR/3 standard*  canteen* NEAR/3 guide*  cafe* NEAR/3 polic*  cafe* NEAR/3 standard*  cafe* NEAR/3 guide*  "school* meal*" NEAR/3 polic*  "school* meal*" NEAR/3 standard*  "school* meal*" NEAR/3 guide*  fundrais* NEAR/3 polic*  fundrais* NEAR/3 standard*  fundrais* NEAR/3 guide* |
| **School** | school* |
