# Supplementary material for: The impact of primary school nutrition policy on the school food environment: a systematic review
Source: Health Promot Int. 2022 Sep 27;37(5):daac084. doi: 10.1093/heapro/daac084 (PMC9514228; doi:10.1093/heapro/daac084)
Supplement: daac084_suppl_Supplementary_Appendix_B [file daac084_suppl_supplementary_appendix_b.docx]

**Appendix B:**

*Quality assessment of included studies against modified Effective Public Health Practice Project Quality Assessment Tool for Quantitative Studies*

| **Authors (year)** | **Selection bias** | **Study design** | **Confounders** | **Data collection methods** | **Withdrawals and dropouts** | **Global rating** |
| --- | --- | --- | --- | --- | --- | --- |
| Behrens et al. 2018 | Moderate | Moderate | Weak | Weak | Weak | Weak |
| Belansky et al. 2013 | Moderate | Weak | N/A | Weak | N/A | Weak |
| Boehm et al. 2020 | Weak | Weak | Weak | Moderate | N/A | Weak |
| Chriqui et al. 2013 | Moderate | Weak | N/A | Moderate | N/A | Moderate |
| Cluss et al. 2014 | Strong | Moderate | N/A | Weak | Strong | Moderate |
| Cummings et al. 2014 | Strong | Moderate | Weak | Weak | Weak | Weak |
| Haroun et al. 2011 | Weak | Moderate | N/A | Weak | Weak | Weak |
| Ishdorj et al. 2016 | Weak | Moderate | Strong | Moderate | Moderate | Moderate |
| Jimenez-Aguilar et al. 2017 | Moderate | Moderate | Weak | Weak | Weak | Weak |
| Kubik et al. 2010 | Moderate | Weak | N/A | Weak | N/A | Weak |
| Long et al. 2010 | Strong | Moderate | Strong | Weak | Weak | Weak |
| Ohri-Vachaspati et al. 2012 | Moderate | Weak | N/A | Weak | N/A | Weak |
| Ohri-Vachaspati et al. 2016 | Weak | Moderate | Strong | Moderate | Moderate | Moderate |
| Patterson et al. 2015 | Weak | Moderate | Strong | Moderate | Weak | Weak |
| Phillips et al. 2010 | Moderate | Weak | N/A | Weak | N/A | Weak |
| Samuels et al. 2010 | Strong | Moderate | N/A | Weak | Strong | Moderate |
| Soares et al. 2017 | Weak | Moderate | Weak | Weak | Weak | Weak |
| Taber et al. 2015 | Moderate | Moderate | Strong | Weak | Moderate | Moderate |
